# Promotive effects of bone morphogenetic protein 2 on angiogenesis in hepatocarcinoma via multiple signal pathways

**DOI:** 10.1038/srep37499

**Published:** 2016-11-25

**Authors:** Wei-han Zuo, Peng Zeng, Xi Chen, Yan-jun Lu, An Li, Jian-bin Wu

**Affiliations:** 1 Department of Oncology, the Second Affiliated Hospital of Nanchang University, Nanchang 330006, China; 2 Department of emergency, the Second Affiliated Hospital of Nanchang University, Nanchang 330006, China; 3 Department of burns, the first Affiliated Hospital, Nanchang University, Nanchang 330006, China

## Abstract

The effects of Bone morphogenetic protein 2 (BMP-2) on the angiogenesis of hepatocellular carcinoma have not yet been observed and its molecular mechanisms is not clear. We first constructed the recombinant lentivirus vectors expressing small hairpin RNA against BMP-2 gene (LV-SH-BMP2) and the recombinant lentivirus vectors over-expressing BMP-2 (overexpression-LV-BMP2), and then the two recombinant lentivirus vectors were respectively transfected into Hep G2 cells. The Hep G2 cells transfected with LV-SH-BMP2 or overexpression-LV-BMP2 were respectively co-cultured with human umbilical vein endothelial cells (HUVECs) to observe the effects of BMP-2 on HUVECs. The effect of BMP-2 on tumor microvessel density (MVD) was examined. The abilities of proliferation, migration and angiogenesis were significantly inhibited in the HUVECs co-cultured with BMP-2 knockdown Hep G2 (all *P* < 0.05), but significantly enhanced in the HUVECs co-cultured with BMP-2 overexpression Hep G2 (all *P* < 0.05). MVD was significantly increased in overexpression-LV-BMP2-transfected Hep G2 tumor, but decreased in LV-SH-BMP2-transfected Hep G2 tumors. The protein expressions of VEGF, p-P38, p-ERK, p-AKT, p-m-TOR were significantly increased after BMP-2 over-expression, or significantly decreased after BMP-2 knockdown (all *P* < 0.05). These results reveal that BMP-2 can enhance HUVEC proliferation, migration and angiogenesis through P38, ERK and Akt/m-TOR pathway.

Primary liver cancer, generally hepatocellular carcinomas (HCCs), is now the second leading cause of death caused by cancers in the world^1^. Angiogenesis, a fundamental hallmark of cancer, is important for the multistep progression of cancer^2^. Bone morphogenetic proteins (BMPs), members of the transforming growth factor-beta family, play an important role in cellular proliferation, differentiation and apoptosis. BMPs, multifunctional regulators, are involved in angiogenesis^3^. BMP-2, a subtype of BMPS, can enhance angiogenic response and stimulate vascularization in tumors. Moreover, BMP-2 inhibitor can be against BMP-2-induced angiogenic response^4^. BMP-2 promotes vascularization and is involved in tumorous angiogenesis possibly through stimulating Id1 and p38 MAPK pathways^5^. BMPR-II knockdown down-regulates VEGF-C expression through MAPK/P38 and MAPK/ERK1/2 pathways^6^. By now, little research has been done about the effects of BMP-2 on invasion, proliferation and angiogenesis of human umbilical vein endothelial cells (HUVECs) and its mechanism. Therefore, in this study, in order to simulate tumor microenvironment, the recombinant lentivirus vectors expressing small hairpin RNA against BMP-2 gene (LV-SH-BMP2) and the recombinant lentivirus vectors over-expressing BMP-2 (overexpression-LV-BMP2) were respectively transfected into Hep G2 cells, and then The Hep G2 cells transfected with LV-SH-BMP2 or overexpression-LV-BMP2 were respectively co-cultured with human umbilical vein endothelial cells (HUVECs) to observe the proliferation, migration and angiogenesis of HUVECs. Additionally, the effect of BMP-2 on tumor microvessel density (MVD) was examined *in vivo*. We also explored some potential pathways through determining protein expressions of VEGF, P38, ERK, PI3K, Akt and mTOR by Western blot after BMP-2 knockdown or over-expression in Hep G2 cells. This study provides a theoretical and experimental basis for exploring the potential mechanism of angiogenesis in human liver cancer.

## Materials and Methods

All study methods were approved by ethics committee of the Second Affiliated Hospital, Nanchang University, and were performed in accordance with relevant guidelines and regulations.

### Chemicals and reagents

Hep G2 cell line and human umbilical vein endothelial cell line were purchased from the Shanghai Mesa Bio-Technology (Shanghai, China). Recombinant LV-SH-BMP2 and overexpression-LV–BMP2 were design by GENECHEM (Shanghai, China). The LV-SH-BMP2-A interference sequence was ACCCTTTGTACGTGGACTT, The LV-SH-BMP2-B interference sequence was GGAGATTCTTCTTTAATTT, The LV-SH-BMP2-C interference sequence was TTCCACCATGAAGAATCTT. The LV-SH-CON sequence was TTCTCCGAACGTGTCACGT. The overexpression-LV-BMP2 was designed according to BMP-2 (NM_001200). The primer 1 was GAGGATCCCCGGGTACCGGTCGCCACCATGGTGGCCGGGACCCGCTG, the primer 2 was TCCTTGTAGTCCATACCGCGACACCCACAACCCTCCAC and the overexpression-LV-CON was empty lentivirus vector. Dulbecco’s Modified Eagle Medium (DMEM) was purchased from HyClone (Logan, UT), Trypsin was purchased from Beijing Solarbio (Beijing, China). Fetal bovine serum was from ExCell Bio (Shanghai, China). BMP-2 antibody and GAPDH antibody were purchased from Proteintech (Chicago, Illinois, USA). P-P38 and p-ERK monoclonal antibodies were purchased from Cell Signaling Biotechnology (Shanghsi, China). Antibodies to VEGF, p-AKT, p-m-TOR were purchased from Abcam Inc (Cambridge, MA, USA).

### Cell culture and lentivirus transfection

Hep G2 cell line and human umbilical vein endothelial cell line were cultured in DMEM supplemented with 10% fetal bovine serum at 37 °C in a fully humidified atmosphere of 5% CO_2._ One day before transfection, Hep G2 cells (2 × 10^5^) were seeded in each well of 6-well plate. The next day, Hep G2 cells cultured in EniS and 10 μg/ml polybrene were infected with different lentivirus vector at 10 MOI, respectively. The CON group only contained the HepG2 which were not transcfected. The LV-SH-BMP2 and overexpression–LV-BMP2 lentivirus were transfected into Hep G2 cells, 12 h later the culture medium was replaced by fresh DMEM medium, and 72 h later the transfection efficiency was measured using a fluorescence microscope. BMP2 levels after LV-SH-BMP2 or overexpression–LV-BMP2 transfection were detected by qPCR and western blot. The groups which were transfected with LV-SH-BMP2 or overexpression-LV-BMP2 lentivirus vectors were set as experimental groups. The untransfected groups were set as control groups.

### Cell co-culture

Six-well Transwell cell culture chamber (Corning) with 0.4 μm pores was used in this study. Hep G2 were incubated in six-well plate, while HUVECs were incubated in upper chamber. ratio of HUVEC: Hep G2 was 1:5. Co-cultures were performed in Dulbecco’s modified Eagle’s medium (DMEM) supplemented with 10% fetal bovine serum.

### Cell proliferation assay

In order to detect the effects of BMP-2 on HUVEC proliferation, LV-SH-BMP2 (LV-SH-BMP2-A, LV-SH-BMP2-B and LV-SH-BMP2-C) and overexpression-LV-BMP2 were transfected into Hep G2 cells. The Hep G2 cells which were not transfected were used as CON groups. HUVECs were co-cultured with different Hep G2 at a ratio of 1:5 for 48 h, and then were used for CCK-8 assay. HUVECs (3 × 10^3^) were seeded in 96-well plate, and then 10 μl of CCK-8 was added to each well followed by incubation for 2 h. The absorbance at 450 nm was measured with a microplate reader.

### Transwell assay

Transwwell assay was performed in 24-well cell culture chambers with 8-μm-sized pores (Corning). After co-cultured with different Hep G2 for 48 h, HUVECs (3 × 10^4^) were placed in the upper surface of the Transwell inserts. The lower chamber contained 600 μl of DMEM supplemented with 1% FBS. Twenty four hours after incubation at 37 °C, the cells not to penetrate the member of the upper chamber were wiped out using cotton swabs. Then the cells to penetrate the member were fixed with methanol and stained with 0.5% crystal violet for 30 min. The cells to penetrate the member were counted in 3 random fields per chamber under an inverted microscope (Olympus), and each experiment was repeated three times

### Scratch test

HUVECs were cultured to confluence on 6-well plates. Then, HUVECs monolayer was scratched horizontally with a 10 μl micropipette tip. Cellular debris was removed by gently washing twice with PBS and then cultured in DMEM supplemented with 1% FBS. Three randomly selected fields along the scraped line were photographed using an inverted microscope. After incubation at 37 °C for 8 h, images were taken and cell migration distances were measured.

### Angiogenic assay

Differentiation of HUVECs was examined by tube formation Matrigel. Matrigel was thawed on ice overnight, spread evenly over each well (50 μL/well) of 96-well plates and polymerized for one hour at 37 °C. HUVECs (6 × 10^4^ cells/well) were plated onto the Matrigel layer and cultured in DMEM supplemented with 1% FBS at 37 °C. Eight hours later, tube formation was observed and captured with a phase contrast microscopy. Total tubular length per well was determined by computer-assisted image analysis using Image Pro Plus software.

### RNA extraction and quantitative real-time PCR (qRT-PCR)

BMP-2 gene expression was examined by quantitative real-time PCR. Total RNA was extracted from Hep G2 with Trizol reagent (Transgen Biotech). Then, total RNA underwent reverse transcription to obtain complementary DNA (cDNA) which was used for the PCR template. The primers of BMP-2 and control GAPDH were synthesized by Invitrogen (Carlsbad, USA). The upstream of BMP-2 primer was 5′- AACCTGCAACAGCCAACT-3′ and downstream was 5′- GCTCAGTGTAGCCCAGGAT-3′ with a length of 418 bp of PCR product. The upstream of control GAPDH primer was 5′- GAAGGTCGGAGTCAACGG-3′ and downstream was 5′- GCTCAGTGTAGCCCAGGAT- 3′ with a length of 825 bp of PCR product. The cycling program was as follows: denaturation at 95 °C for 30 seconds; denaturing at 95 °C for 5 s, annealing at 60 °C for 30 s, 40 cycles.

### Western blot assay

Pre-cooled lysis buffer at 4 °C (volume of five times) was added in differently cultured cells to extract protein. The protein was stored at −20 °C for future use. Protein concentration was determined using BCA method. Protein and buffer were mixed at a ratio of 5:1, and then placed in boiling water for 5–10 min. A total of 80 μg of sample per well underwent 12% SDSPAGE, and then was transferred onto nitrocellulose membrane followed by sealing using 10 ml of TBST containing 0.5% dried skim milk at 4 °C overnight. Following washing two times using TBST with each time for several seconds, the antibodies of BMP-2, VEGF, p-PI3K, PI3K, p-AKT, AKT, p-m-TOR and m-TOR were diluted according to the manufacturer’s instructions and incubated overnight at 4 °C. Next day, following washing three times using TBST with each time for 10 min, HRP-labeled secondary antibody were added for 2 h. The mixture was washed three times using TBST with each time for 10 min followed by visualization using substrate electrochemiluminescence.

### Transplanted tumor mouse model

Twenty 6 to 8-week-old female mice weighing 18–22 g, were randomly divided into five groups: the group of LV-SH-BMP2-transfected Hep G2, the group of overexpression-LV-BMP2-transfected Hep G2, the group of LV-SH-CON-transfected Hep G2, the group of overexpression-LV-CON-transfected Hep G2 and the group of untransfected Hep G2 (CON). The mice were administered above Hep G2 cells (5 × 10^6^) in single suspension/mouse via a subcapsular injection under the right armpit. Fourteen days later, the mice were killed and the tumor tissues were removed and fixed with 4% paraformaldehyde, and paraffin-embedded tissues were cut into slices for immunohistochemistry.

### Microvessel density (MVD)

The tissue sections were dewaxed with xylene, and the slides were then soaked in graded concentrations of alcohol (100%, 95%, 85% and 70%) for 5 min, stained with hematoxylin for 3 min and eosin for one minute. Vascular endothelial cells were marked with CD34 using an EnVision™ detection kit following the manufacturer’s protocols. According to the method raised by Weidner, areas with the most intense coloring were selected as ‘hot spots’, and the microvessels were counted under the microscope and imaged. For one slice, the microvessels in 3 ‘hot spots’ were counted and their average was serves as the slice MVD.

### Statistical analysis

All experiments were performed at least three times. Data are expressed as mean values ± SD. The statistical analyses were performed using Student’s *t* test for comparisons to control and one-way ANOVA for comparisons between multiple groups. Statistical significance was established at *P* < 0.05.

## Result

### Expressions of BMP-2 mRNA and protein after BMP-2 silence and over-expression in HepG2 cells

**Figure 1:**
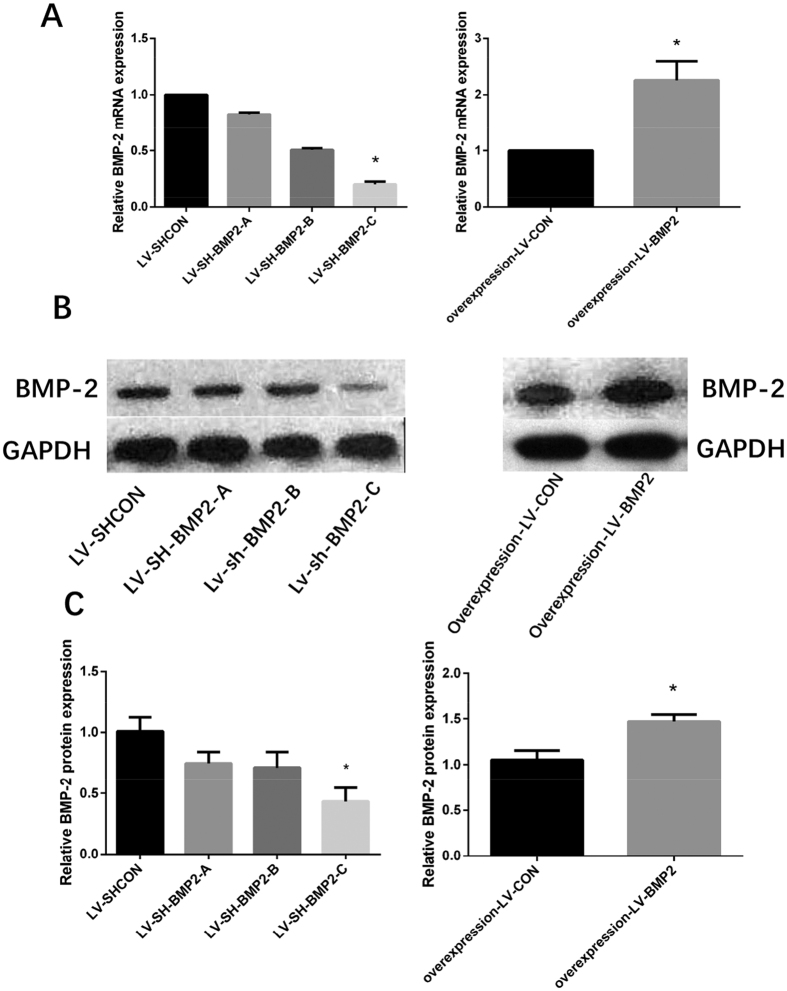
Expressions of BMP-2 mRNA and protein in HepG2 cells. (**A**) BMP-2 mRNA expression level is significantly lower in LV-SH-BMP2-C group than in LV-SH-BMP2-A and in LV-SH-BMP2-B groups. BMP-2 mRNA expression level is significantly higher in overexpression-LV-BMP2 group than in overexpression-LV-CON group. (**B**) Results of BMP-2 protein expression detected by western blot analysis in different groups (**C**) BMP-2 protein expression level is significantly lower in LV-SH-BMP2-C group than in LV-SH-BMP2-A and in LV-SH-BMP2-B groups. BMP-2 protein expression level is significantly higher in overexpression-LV-BMP2 group than in overexpression-LV-CON group. Notes: BMP-2: Bone morphogenetic protein 2; LV-SH-BMP2: lentivirus vectors expressing small hairpin RNA against BMP-2 gene. *Indicates *P* < 0.05 as compared with other groups.

The expressions of BMP-2 mRNA and protein in HepG2 cells were detected by qPCR and western blot analysis. The expressions of BMP-2 mRNA and protein were significantly lower in the LV-SH-BMP2-C group (*P* < 0.05) as compared with LV-SH-BMP2-A and LV-SH-BMP2-B groups. Therefore, we selected the LV-SH-BMP2-C group as the down-regulation group (LV-SH-BMP2). Compared with the over-expression LV-CON group, the expressions of BMP-2 mRNA and protein were significantly higher in the over-expression LV-BMP2 group (*P* < 0.05) (Fig. 1).

### Effects of BMP-2 on the proliferation of co-cultured HUVECs

**Figure 2:**
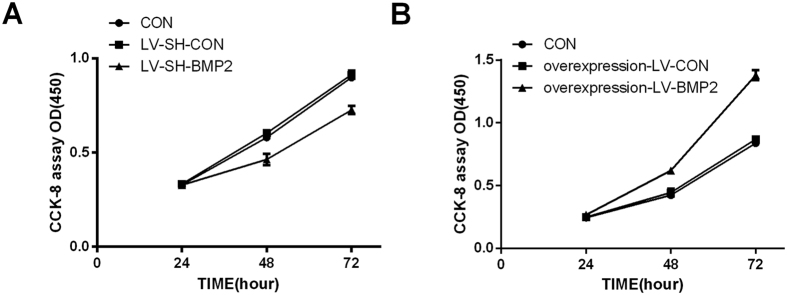
HUVEC proliferation. (**A**) HUVEC proliferation is significantly inhibited in LV-SH-BMP2 group as compared with those in LV-SH-CON and CON groups (all *P* < 0.05). But no significant difference is found between LV-SH-CON and CON groups in HUVEC proliferation (*P* > 0.05). HUVEC proliferation is significantly inhibited in LV-SH-BMP2 group as compared with those in LV-SH-CON and CON groups (all *P* < 0.05). (**B**) HUVEC proliferation is significantly higher in overexpression–LV-BMP2 group than in overexpression–LV-BMP2 and CON groups (all *P* < 0.05). Notes: BMP-2: Bone morphogenetic protein 2; HUVEC: human umbilical vein endothelial cell; LV-SH-BMP2: lentivirus vectors expressing small hairpin RNA against BMP-2 gene.

In order to detect the effects of BMP-2 on HUVEC proliferation, Hep G2 cells were transfected with different lentivirus (LV-SH-CON, LV-SH- BMP2, overexpression-LV-CON, overexpression-LV-BMP2). The untransfected Hep G2 was used as CON. Both transfected Hep G2 cells and untransfected Hep G2 cells were respectively co-cultured with HUVECs at a ratio of 1: 5 for 48 h, and then these HUVECs were used to detect HUVEC proliferation. We found that HUVEC proliferation was inhibited in the Hep G2 cells with BMP-2 silence; while in the Hep G2 cells with BMP-2 over-expression, HUVEC proliferation was enhanced (Fig. 2).

### Effects of BMP-2 on the migration of co-cultured HUVECs

**Figure 3:**
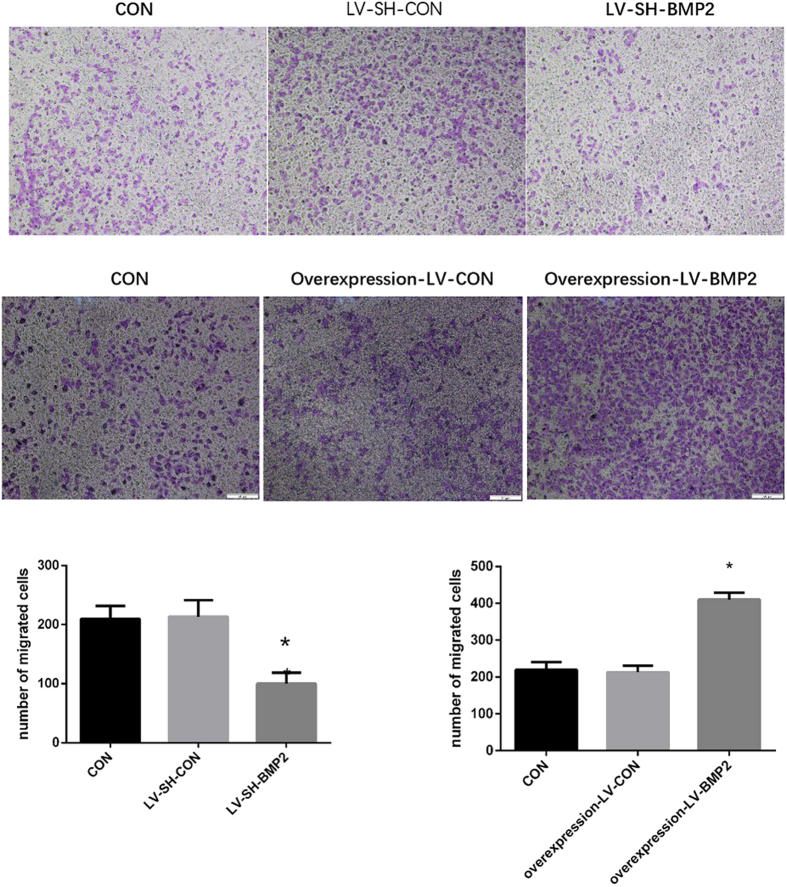
Transwell assay. The migrated number of HUVECs co-cultured with the LV-SH-BMP2-transfected Hep G2 is significantly decreased as compared with those of HUVECs co-cultured with LV-SH-CON-transfected Hep G2 or untransfected Hep G2 (CON) (all *P* < 0.05). By contrast, HUVEC migratory ability is significantly enhanced after co-cultured with overexpression-LV-BMP2 Hep G2 (*P* < 0.05). Notes: BMP-2: Bone morphogenetic protein 2; HUVEC: human umbilical vein endothelial cell; LV-SH-BMP2: lentivirus vectors expressing small hairpin RNA against BMP-2 gene. *Indicates *P* < 0.05 as compared with other groups.

HUVEC migratory ability was evaluated by transwell assay and scratch test. The migrated number of HUVECs co-cultured with the LV-SH-BMP2-transfected Hep G2 was significantly decreased as compared with those of HUVECs co-cultured with LV-SH-CON-transfected Hep G2 or untransfected Hep G2 (CON) (all *P* < 0.05). However, a difference was not significant in the migrated number between the HUVECs co-cultured with untransfected Hep G2 (CON) and LV-SH-CON–transfected Hep G2 (*P* > 0.05). By contrast, HUVEC migratory ability was significantly enhanced after co-cultured with overexpression-LV-BMP2 Hep G2 (*P* < 0.05). However, a difference was not significant in the migrated number between the HUVECs co-cultured with untransfected Hep G2 (CON) and overexpression-LV-CON- transfected Hep G2 (*P* > 0.05) (Fig. 3).

**Figure 4:**
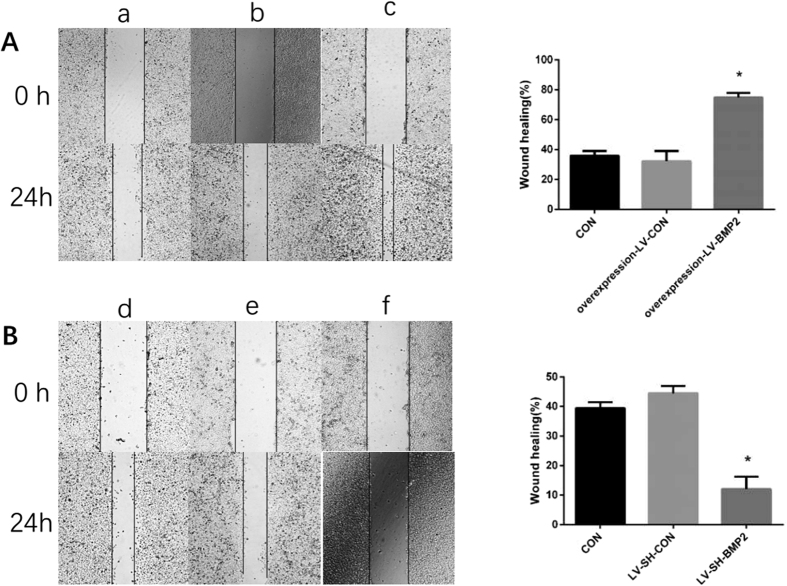
Scratch test. a. HUVECs co-cultured with untransfected HepG2 b. HUVECs co-cultured with overexpression–LV-CON transfected HepG2 c. HUVECs co-cultured with overexpression–LV-BMP2 HepG2 d. HUVECs co-cultured with untransfected HepG2 e. HUVECs co-cultured with LV-SH-CON HepG2 f. HUVECs co-cultured with LV-SH-BMP2 HepG2 Notes: BMP-2: Bone morphogenetic protein 2; HUVEC: human umbilical vein endothelial cell; LV-SH-BMP2: lentivirus vectors expressing small hairpin RNA against BMP-2 gene. *Indicates *P* < 0.05 as compared with other groups.

In scratch test, the number of HUVECs, co-cultured with LV-SH-BMP2-transfected Hep G2 and migrating to the open area, was less; and its scratch was wider as compared with those of HUVECs co-cultured with LV-SH-CON-transfected Hep G2 or untransfected Hep G2 (CON) (all *P* < 0.05). By contrast, the scratch of HUVECs co-cultured with overexpression-LV-BMP2 Hep G2 was significantly thinner as compared with those of HUVECs co-cultured with untransfected Hep G2 (CON) or overexpression-LV-CON-transfected Hep G2 (all *P* < 0.05). However, there was not a significant difference in the scratch between HUVECs co-cultured with untransfected Hep G2 (CON) and overexpression-LV-CON-transfected Hep G2 (P > 0.05) (Fig. 4).

### Effects of BMP-2 on the angiogenesis of co-cultured HUVECs

**Figure 5:**
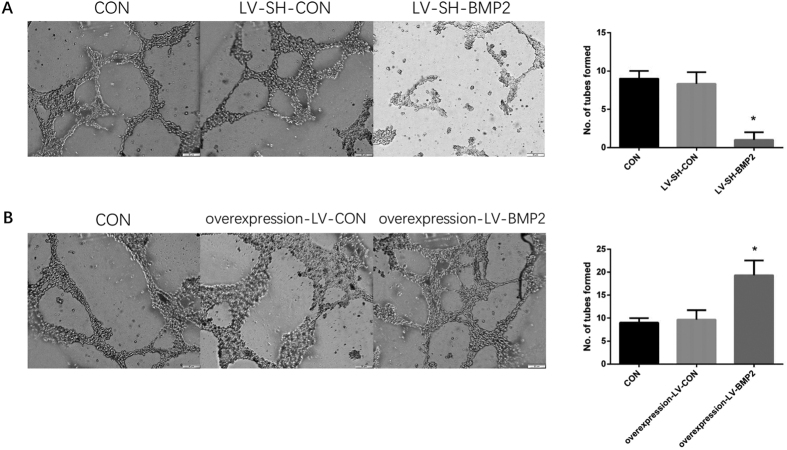
Angiogenic assay. (**A**) The angiogenesis of HUVECs is significantly decreased after co-cultured with LV-SH-BMP2-transfected HepG2 as compared with those in CON group and LV-SH-CON group. (**B**) After co-cultured with overexpression-LV-BMP2 Hep G2, the angiogenesis of HUVECs is significantly enhanced as compared with those in CON group and overexpression-LV-CON group. Notes: BMP-2: Bone morphogenetic protein 2; HUVEC: human umbilical vein endothelial cell; LV-SH-BMP2: lentivirus vectors expressing small hairpin RNA against BMP-2 gene. *Indicates *P* < 0.05 as compared with other groups.

In angiogenic assay, the angiogenic ability was significantly lower and the number of tube formation was significantly less in the HUVECs co-cultured with LV-SH-BMP2-transfected Hep G2 than in HUVECs co-cultured with untransfected Hep G2 (CON) or LV-SH-CON-transfected Hep G2 (all *P* < 0.05). However, after co-cultured with overexpression-LV-BMP2 Hep G2, the angiogenesis of HUVECs was significantly enhanced as compared with CON group and overexpression-LV- CON group. Also, the difference was not significant between CON group and overexpression-LV-BMP2 group (P > 0.05) (Fig. 5).

### MVD in tumor tissue of mice

**Figure 6:**
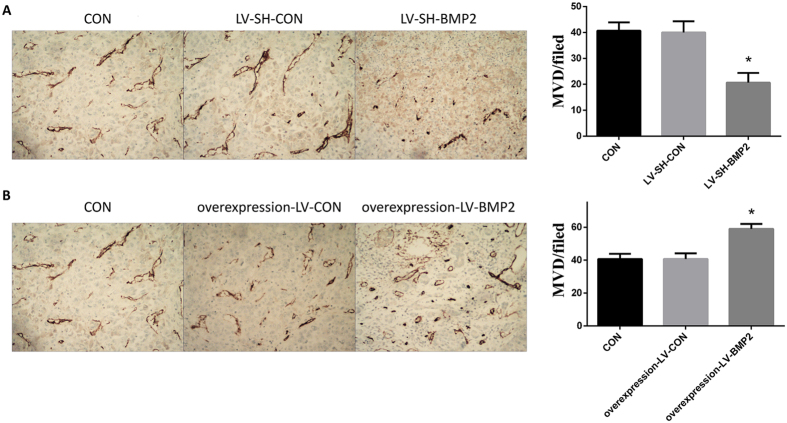
Assay for microvessel density. (**A**) the MVD in tumor tissue is significantly decreased in mice of LV-SH-BMP2 group as compared with those in CON group and LV-SH-CON group. (**B**) the MVD in tumor tissue is significantly increased in mice of overexpression-LV-BMP2 group as compared with those in CON group and overexpression-LV-CON group. Notes: BMP-2: Bone morphogenetic protein 2; MVD: microvessel density; LV-SH-BMP2: lentivirus vectors expressing small hairpin RNA against BMP-2 gene. *Indicates *P* < 0.05 as compared with other groups.

In this assay, compared with the untransfected Hep G2 (CON) and LV-SH-CON-transfected Hep G2 groups, the MVD in tumor tissue was significantly decreased in the mice of LV-SH-BMP2-transfected Hep G2 group, whereas it was significantly increased in the mice overexpression-LV-BMP2-transfected Hep G2 group (all *P* < 0.05). However, the MVD in tumor tissue was not significantly different between CON group and either LV-SH-CON group or overexpression-LV-CON group (all *P* > 0.05) (Fig. 6)

### Effects of BMP-2 on the expressions of VEGF, p-P38, p-ERK, p-AKT and p-m-TOR

**Figure 7:**
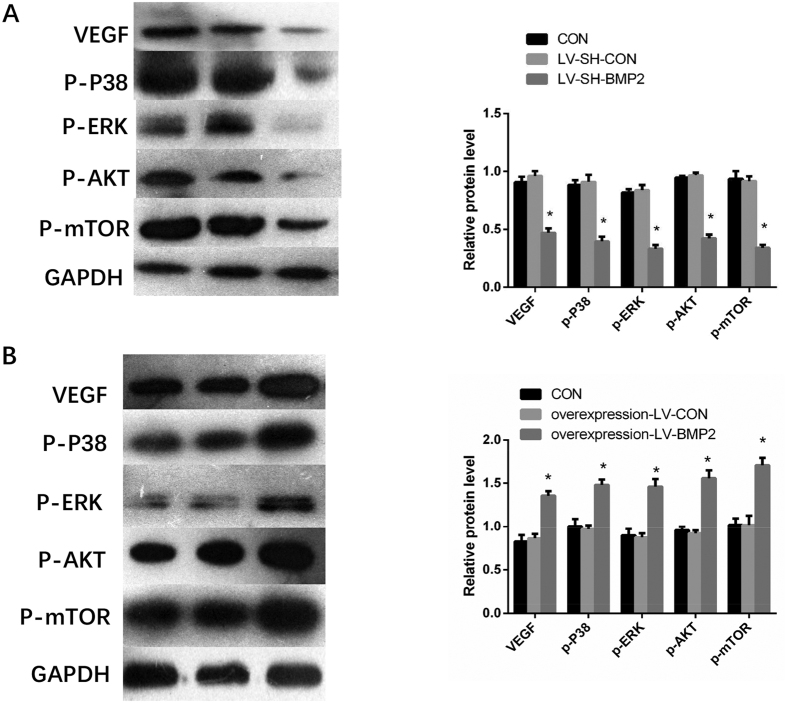
Expressions of VEGF, p-P38, p-ERK, p-AKT and p-m-TOR detected by Western blot. (**A**) BMP2 silence down-regulates the expression of VEGF, p-P38, p-ERK, p-AKT and p-m-TOR expressions (**B**) The protein expressions of VEGF, p-P38, p-ERK, p-AKT and p-m-TOR are up-regulated in overexpression-LV-BMP2 group Notes: BMP-2: Bone morphogenetic protein 2; VEGF: vascular endothelial growth factor * Indicates *P* < 0.05 as compared with other groups.

Western blot revealed a significant up-regulation of VEGF, p-P38, p-ERK, p-AKT and p-m-TOR expression in overexpression-LV-BMP2 group. However, BMP-2 silence lead to a down-regulation of VEGF, p-P38, p-ERK, p-AKT and p-m-TOR expressions. Compared with CON group, protein expression did not show significant differences in LV-SH-CON and overexpression-LV-CON groups (Fig. 7).

## Discussion

BMPs not only regulate the formation of bone and cartilage, but also play a vital role in tumor’s proliferation, invasion and angiogenesis. As one branch of BMPs, BMP-2 also has important effects on tumor’s proliferation, invasion and angiogenesis. Kim *et al*.^7^ found that BMP-2 could enhance the motility and invasiveness of colon cancer cells through up-regulation of cancer stem cells (CSCs) and played an important role in the metastasis of colon cancer. BMP-2 knockdown in lung cancer cell lines A549 and H460 suppressed their proliferation and migration^8^. BMP2/BMPR axis triggered Epithelial mesenchymal transition (EMT) process in gastric cancer, and then was involved in tumor cell migration, invasion and metastasis via the activation of PI3K/AKT and MEK/ERK pathways^9^. BMP-2 promotes motility and invasion of gastric cancer cells by activating PI-3 kinase/Akt^10^. Therefore, these data indicate that BMP-2 has important biological activity in the process of tumor’s formation and development.

It is widely known that angiogenesis plays an important role in malignant tumor’s occurrence, development, invasion and metastasis. It has been reported that BMP-2 promotes angiogenesis by stimulating VEGF secretion^11^. BMP-2 can enhance neovascularization in progressive lung tumor^4^. The two angiogenic factors, VEGF and BMP-2, play important roles in the biological assessment for lung malignancy^12^. Bai Y *et al*.^13^ have reported that BMP-2 can amplify the effects of VEGF and FGF-2 on angiogenic activity, and the ternary combination of BMP-2, VEGF and FGF-2 exhibits a positive and synergistic effect on HUVECs angiogenesis. Therefore, these data above demonstrate the role of BMP-2 in tumor’s angiogenesis.

PI3K/AKT/m-TOR signaling pathway, participating in tumor’s progression, plays an important role in cellular growth, invasion and apoptosis. It is reported that LZTS1 could inhibit HCC cell proliferation by impairing PI3K/Akt pathway^14^. ANT2 suppression down-regulates PI3K/Akt signaling pathway, resulting in HCC inhibition^15^. VEGF is known to be the strongest pro-angiogenic factor, VEGF silence can inhibit HCC progression through PI3K/AKT signaling pathway^16^. VEGF silencing suppresses cell proliferation, promotes cell apoptosis and reduces angiogenesis in osteosarcoma through inactivating PI3K/AKT signaling pathway^17^. PI3K/AKT signaling pathway also plays an important role in regulating vasculature and angiogenesis^18^.

In previous study, we found that BMP-2 silence could inhibit liver cancer cell’s proliferation, migration and invasion by down-regulation of MMP2 and MMP9 through MAPK/ERK pathway^19^. Another study has shown that siRNA targeting BMPR-II can significantly inhibit HepG2 proliferation and invasion, promote apoptosis and block HepG2 in S phase by down-regulating VEGF-C expression through MAPK/P38 and MAPK/ERK1/2 pathways, especially MAPK/P38^6^. However, it has been unclear whether BMPs directly promote vascular endothelial cell proliferation and its mechanism in liver cancer, and moreover little research has been done regarding the role of BMP-2 in HCC angiogenesis. Therefore, in this study, we used recombinant lentivirus vector to silence and over-express BMP-2 in Hep G2 cell line, and then HUVECs were used to co-culture with these Hep G2. Forty eight hours after incubation, we detect HUVEC’s proliferation, migration and tubulogenesis ability respectively. In order to explore its underline mechanism, we also observed the changes in VEGF, p-P38, p-ERK, p-AKT and p-m-TOR. Our results revealed that the HUVEC’s proliferation, migration and angiogenesis ability were enhanced after co-cultured with Hep G2 which was infected with overexpression BMP-2 lentivirus vector. However, HUVEC’s proliferation, migration and angiogenesis were significantly inhibited after co-cultured with BMP2-silenced Hep G2 cells. Similarly, our MVD assay proved that the overexpressed-BMP-2 could enhance angiogenesis ability of Hep G2 cells *in vivo*. On the contrast, the knockdown of BMP-2 could decrease angiogenesis ability of Hep G2 cells in mice. We also found that after BMP-2 knockdown, AKT/m-TOR signal pathway were significantly down-regulated and p-P38, p-ERK, VEGF protein levels were also down-regulated. By contrast, BMP-2 over-expression up-regulated VEGF, p-P38, p-ERK and AKT/m-TOR signal pathway. These data suggest that BMP-2 promotes the proliferation and migration of vascular endothelial cells, inducing tumor angiogenesis.

In summary, these data show that BMP-2 promotes HUVEC’s proliferation, migration and angiogenesis and induces VEGF expression through the Akt/m-TOR and P38,ERK signaling pathways. Thereby, BMP-2 may be a novel anti-angiogenesis therapeutic target for HCC.

## Additional Information

**How to cite this article**: Zuo, W.-h. *et al*. Promotive effects of bone morphogenetic protein 2 on angiogenesis in hepatocarcinoma via multiple signal pathways. *Sci. Rep*. **6**, 37499; doi: 10.1038/srep37499 (2016).

**Publisher’s note:** Springer Nature remains neutral with regard to jurisdictional claims in published maps and institutional affiliations.
